# Clearance of Asymptomatic *P. falciparum* Infections Interacts with the Number of Clones to Predict the Risk of Subsequent Malaria in Kenyan Children

**DOI:** 10.1371/journal.pone.0016940

**Published:** 2011-02-24

**Authors:** Anne Liljander, Philip Bejon, Jedidah Mwacharo, Oscar Kai, Edna Ogada, Norbert Peshu, Kevin Marsh, Anna Färnert

**Affiliations:** 1 Infectious Diseases Unit, Department of Medicine Solna, Karolinska Institutet, Stockholm, Sweden; 2 Centre for Geographical Medicine Research (Coast), Kenya Medical Research Institute, Kilifi, Kenya; 3 Nuffield Department of Clinical Medicine, Oxford University, John Radcliffe Hospital, Oxford, United Kingdom; Instituto Gulbenkian de Ciência, Portugal

## Abstract

**Background:**

Protective immunity to malaria is acquired after repeated infections in endemic areas. Asymptomatic multiclonal *P. falciparum* infections are common and may predict host protection. Here, we have investigated the effect of clearing asymptomatic infections on the risk of clinical malaria.

**Methods:**

Malaria episodes were continuously monitored in 405 children (1–6 years) in an area of moderate transmission, coastal Kenya. Blood samples collected on four occasions were assessed by genotyping the polymorphic *P. falciparum* merozoite surface protein 2 using fluorescent PCR and capillary electrophoresis. Following the second survey, asymptomatic infections were cleared with a full course of dihydroartemisinin.

**Results:**

Children who were parasite negative by PCR had a lower risk of subsequent malaria regardless of whether treatment had been given. Children with ≥2 clones had a reduced risk of febrile malaria compared with 1 clone after clearance of asymptomatic infections, but not if asymptomatic infections were not cleared. Multiclonal infection was associated with an increased risk of re-infection after drug treatment. However, among the children who were re-infected, multiclonal infections were associated with a shift from clinical malaria to asymptomatic parasitaemia.

**Conclusion:**

The number of clones was associated with exposure as well as blood stage immunity. These effects were distinguished by clearing asymptomatic infection with anti-malarials. Exposure to multiple *P. falciparum* infections is associated with protective immunity, but there appears to be an additional effect in untreated multiclonal infections that offsets this protective effect.

## Introduction

Immune protection against *Plasmodium falciparum* malaria is gradually acquired after repeated infections and largely dependent on exposure intensity [1]. The severity and frequency of malaria episodes decrease with age in areas of high transmission. Nonetheless, parasites are often detected in the blood of asymptomatic individuals and parasite prevalence is widely used as a measure of transmission level [2]. Although, the mechanisms by which protective malaria immunity is acquired and maintained are still incompletely understood, antibodies are recognized to be important [3]. Naturally acquired antibody responses to malaria in younger children may, however, be rather short lived [4], [5]. Although the persistence of asymptomatic parasitemia reflects the non-sterilizing nature of malaria immunity, parasites may also be important for the maintenance of immune responses [6] and protection against new infections [7].

A characteristic of *P. falciparum* infections is the presence of several genetically distinct parasite subpopulations, i.e. clones, within a single individual. The number of infecting clones varies with age [8], [9] and transmission intensity [8], [10], [11], . Moreover, asymptomatic multiclonal infections have been correlated with varying risks of developing clinical malaria depending on the transmission intensity. In high transmission settings and in older children, a high diversity has been associated with a reduced risk of subsequent clinical malaria [13], [14]. In contrast, in areas of low transmission and in younger children, a high number of clones are associated with a higher risk [15], [16], [17].

To clarify the importance of persistent multiclonal *P. falciparum* infections on host immunity, we have investigated how clearance of asymptomatic infections affects the subsequent risk of clinical malaria in children (1–6 years) living on the coast of Kenya. A short acting anti-malarial drug (dihydroartemisinin) was given at the beginning of the malaria transmission season [18]. The diversity of *P. falciparum* infections was investigated by genotyping the highly polymorphic gene encoding the merozoite surface protein 2 (*msp2*) using fluorescent PCR and capillary electrophoresis [19]. The use of a short acting drug, with no or minor prophylactic effect allowed for the specific assessment of clearance of parasites without affecting exposure. Further, the heterogeneity of exposure was addressed to distinguish the effect of protection due to immunity from that of lack of exposure [20].

## Materials and Methods

### Study design

The study included samples collected during a randomized controlled trial of a candidate malaria vaccine. The study and extended follow up has been described in detail elsewhere [18], [21]. Briefly, 405 children aged 1–6 years living in Junju sublocation in Kilifi District, coastal Kenya were included. Malaria transmission is moderate in Junju, with an estimated entomological inoculation rate (EIR) of 22–53 in 1998 [22] and 19.8 infective bites per person per year (CI 10.35–38.23) in 2010 (Janet Midega, personal communication). Peak malaria transmission is associated with the two rainy seasons in May to July and in November, but low level transmission occurs all year. The five study villages can further be divided into “high” (n = 2) and “low” (n = 3) transmission villages based on previous surveillance of re-infection rates [23]. Written consents were obtained from parents or guardians of participating children. Ethical approval was obtained from Kenyan Medical Research Institute National Ethics Committee, the Central Oxford Research Ethics Committee, the London School of Hygiene and Tropical Medicine Ethics Committee and from the Regional Ethical Review Board in Stockholm, Sweden.

### Clearing of asymptomatic infections and cross-sectional blood samples

Scheduled venous blood samples were collected in EDTA at initial screening in February 2005 and post-vaccination in May 2005. All children were then treated (independently of being parasite positive or not) with direct observed dihydroartemisinin monotherapy for seven days to clear asymptomatic parasiteamias, beginning on the day of the blood sample taken in May. Parasite clearance was confirmed by microscopical analysis of blood films one week after ended treatment. Additional blood samples were taken at cross-sectional surveys in August 2005 and January 2006 followed by an additional 9 months follow-up. To calculate parasite density, the number of asexual-stage parasites per 200 leukocytes was counted by conventional light microscopy, and parasiteamia was estimated on the basis of an assumed uniform white cell count of 8000 leukocytes/µl blood. Hemoglobin (Hb) levels were measured at all cross-sectional surveys.

### Monitoring for clinical malaria

Clinical malaria was monitored weekly by home visits during the total 18 months follow-up. Fieldworkers were resident in the villages where children were recruited and readily accessible for unscheduled visits if the children developed fever. When the temperature was greater than 37.5°C, a blood film and a rapid diagnostic test (OptiMAL®, DiaMED) for malaria were conducted. The blood films were reviewed within 24–48 hours, but the immediate treatment decisions were based on the rapid test result.

### Clinical case definitions

Clinical malaria was defined as fever (axillary temperature >37.5°C) and presence of >2500 *P. falciparum* parasites/µl. This parasite threshold was set to increase the specificity of the endpoint since asymptomatic parasiteamias are common among children in this endemic area [24]. All clinical malaria episodes (independent of parasite densities) were treated with artemether-lumefantrine (CoArtem®) according to Kenyan national guidelines.

### *Msp2* genotyping of *P. falciparum* infection

Blood samples (n = 1342) collected at the four cross-sectional surveys were genotyped for *msp2*. Blood smear positive and negative samples were included, so as to include submicroscopic infections. DNA was extracted using ABI Prism 6100 Nucleic Acid PrepStation (Applied Biosystems) (n = 858) or PUREGENE™ DNA Isolation Kit (Gentra systems) (n = 484). *P. falciparum* parasite populations were characterized by *msp2* genotyping using fluorescent PCR and capillary electrophoresis (CE) as described previously [19]. Briefly, the entire polymorphic region of *msp2* (block 3) was amplified in a primary reaction, followed by nested reactions where the respective allelic types of *msp2*, FC27 and IC, were targeted with fluorescently labeled primers in separate reactions. Fragment analysis was performed in 96-well format in a 3130xl DNA sequencer and the results were analyzed using GeneMapper® Software version 4.0 (both from Applied Biosystems). A 150 relative fluorescent unit (rfu) cut off was set to distinguish true fragment peaks from background fluorescence and other artefacts. Microscopy positive samples that were negative by PCR were concentrated by ethanol precipitation before repeating the PCR reaction.

### Statistical methods

Analysis was performed using STATA (v10). Only strictly asymptomatic children were included in the analysis, therefore all children with a clinical episode at survey or in the period 28 days before until 7 days after survey were excluded. Each child contributed up to 4 separate periods of three months follow-up after the respective cross-sectional bleeds.

Association between number of clones (no infection/1 clone/2 or more clones) and village of residence (high/low transmission), age (years, continuous variable), Hb levels, vaccine i.e. malaria or control (rabies vaccine) and insecticide treated bednet (ITN) use (yes/no) were investigated by a Poisson model adjusted for transmission season (dry/rainy) and corrected for repeated observations per child by the robust cluster estimator. A single multivariable model was produced. Correlation between parasite density and age and number of clones was estimated using Spearman rank correlation coefficient. This method was also used to calculate the correlation of number of clones between surveys.

For survival analyses, data from the respective follow-up periods was analyzed individually followed by pooled analysis for the surveys without treatment (i.e. surveys 1, 3 and 4). The follow-up after survey 2, with treatment, was analyzed separately. Cox regression was used to assess the time to the first or only clinical episode during follow-up (without/with treatment) in relation to number of clones (0, 1 and ≥2). Hazard ratios (HR) were adjusted for ITN use, age and village of residence (high/low transmission) and transmission season i.e. dry/rainy (applicable only in the pooled analysis). Schoenfeld residuals for non-proportionality were analysed for each covariate. The linear fit of the model was tested by examining multiple fractional polynomials and was not rejected in favour of polynomial fits for age (*P* = 0.38). MOI was fit in categories (0 clones/1 clone/≥2 clones).

In an analysis taking exposure into account, survey 2 was used as baseline and the outcome during the three months follow-up after parasite clearance was classified into three categories; (1) clinical malaria (fever >37.5°C and >2500 *P. falciparum* parasites/µl) during follow-up, (2) no clinical malaria but presence of asymptomatic parasiteamia at the cross-sectional survey three months later (survey 3), or (3) remaining uninfected i.e. having no clinical episode during follow-up nor any detectable parasites at the following cross-sectional survey. Factors associated with the different outcomes, i.e. number of clones, age, village of residence (high/low transmission) and ITN use, were investigated by logistic regression. When the outcome was re-infection (i.e. clinical malaria or asymptomatic parasiteamia), the analysis was performed excluding children who remained uninfected as these children were considered less exposed.

## Results

Out of the 405 children, *msp2* genotyping data was available for 337, 360, 360 and 288 children from the four respective cross-sectional surveys. Data records from 71 children were excluded due to anti-malarial intake within 28 days before until 7 days after survey.

Vaccination had no effect on the incidence of clinical malaria episodes or on prevalence of asymptomatic parasiteamias [18], nor on number of clones measured in this study (*P = 0.9 by Student's T*). The vaccine groups, i.e. malaria/control were therefore pooled for further analysis.

### Parasite prevalence and densities

At the first cross-sectional survey (Feb 2005), 263 (78%) children were positive for *P. falciparum* by PCR. Parasite prevalence and densities were lower at the next survey just before drug treatment (May 2005) as well as after the two consecutive surveys three (Aug 2005) and nine months (Jan 2006) after treatment (Table 1). *P. falciparum* infections were more often detected by PCR than by microscopy at all time points, with an additional 5.9%–10.8% being positive by PCR (Table 1). The prevalence of asymptomatic infections increased with age (odds ratio OR = 1.11 per year, 95% confidence interval CI 1.07–1.16 by logistic regression) and was associated with village of residence (OR = 1.18 95% CI 1.03–1.33 by logistic regression in the high compared to the low transmission villages). There was a linear correlation between parasite densities and age (correlation coefficient, r = 0.13 *P<0.001*).

**Table 1:** Parasitological characteristics at three-monthly cross-sectional surveys.

		Before treatment		After treatment	
		Survey 1 (Feb 2005)	Survey 2 (May 2005)	Survey 3 (Aug 2005)	Survey 4 (Jan 2006)
Parasite prevalence, % (95% CI)	microscopy	72.1 (67–77)	48.2 (43–53)	29.1 (24–34)	32.9 (27–38)
	PCR	78.0 (74–83)	59.0 (53–64)	37.5 (32–43)	39.8 (34–45)
Parasite density^a^, mean (95% CI)		708.0 (547.0–920.5)	223.9 (180.7–283.8)	112.2 (90.6–140.6)	120.2 (97.7–154.5)

a In microscopy positive children.

Anti-malarial treatment was given to clear all asymptomatic parasiteamias just after survey 2.

### Infection diversity at the cross-sectional surveys

**Figure 1:**
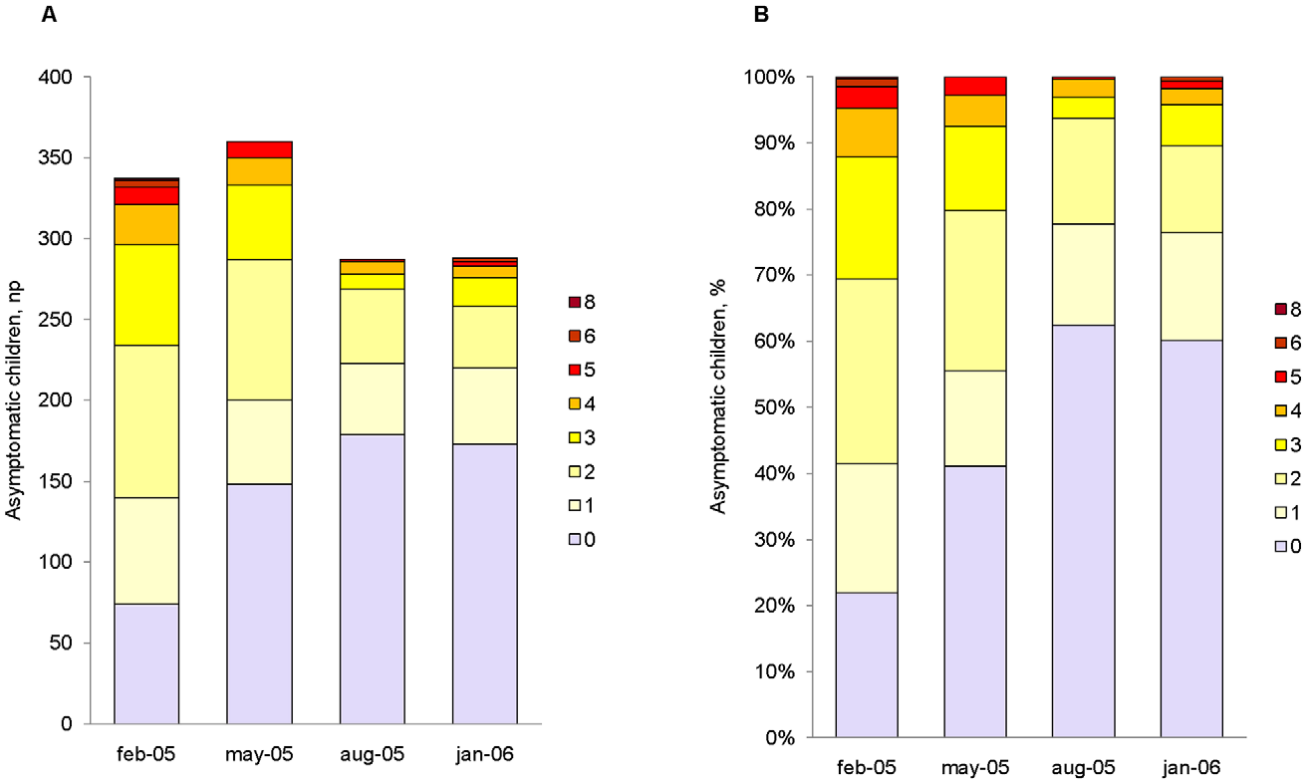
Number (A) and proportion (B) of asymptomatic children infected with different number of *msp2* clones.

**Figure 2:**
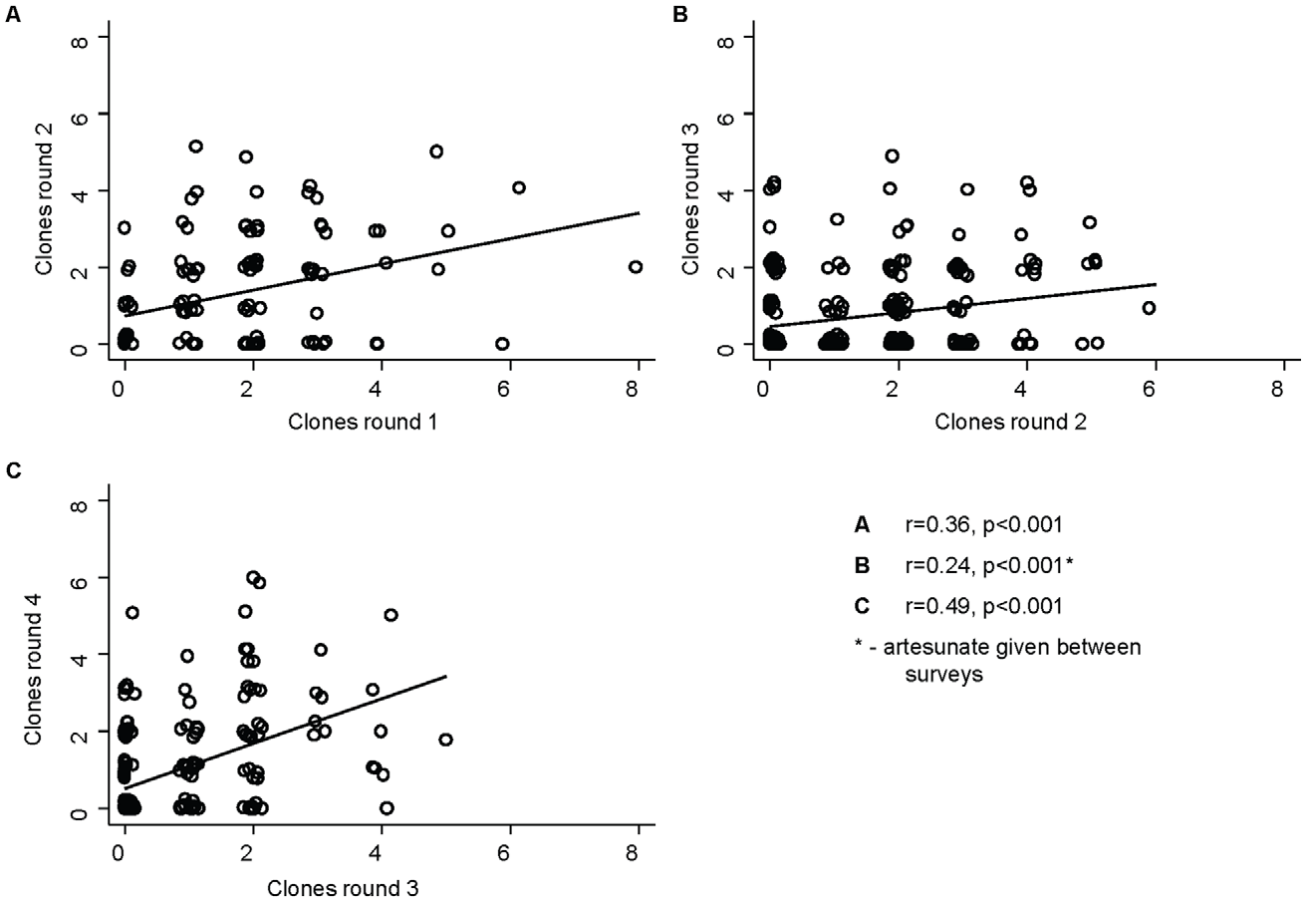
Intra-individual correlation in number of infecting clones between surveys without (A and C) and with treatment (B).

*P. falciparum* infections were mainly composed of ≥2 clones with up to 8 clones at the most (Figure 1). The proportion of infections with multiple clones was higher in the two first surveys (75% and 76%) than in the two last surveys after treatment was given (59.3% and 59.1%), respectively. There was a high intra-individual consistency in the number of clones between the surveys without treatment. Individuals had a similar number of clones in these consecutive pairs of surveys with a Spearman rank correlation coefficient (r) of 0.39 (*P<0.001*) for survey 1 and 2, and r = 0.53 (*P<0.001*) for survey 3 and 4, respectively (Figure 2, Table S1).

In contrast, the correlation was not as high when comparing survey 2 and 3, (r = 0.21, *P<0.001*) with an overall lower number of clones in the survey after treatment (Figure 2, Table S1).

At all surveys, the number of clones was associated with age (incidence risk ratio IRR = 1.17, 95% CI 1.11–1.23 for each year of age by Poisson regression *P*<0.0005), village of residence (IRR = 0.83, 95% CI 0.71–0.99 in the low transmission villages compared to the high transmission, *P* = 0.032) and Hb levels (IRR = 0.9, 95% CI 0.87–0.94 per g/dl increase *P*<0.0005), however not with ITN use (IRR = 0.88, 95% CI 0.73–1.04, *P* = 0.14). There was no systematic difference in the results obtained with the two different DNA extraction methods (data not shown). The number of clones was correlated with age (Spearman non-parametric correlations r = 0.21, *P*<0.001) (Figure S1). Parasite densities were correlated with the number of clones (Spearman non-parametric correlation coefficient r = 0.78, *P<0.001*). This correlation was non-linear indicating that at high parasite densities, further increases in density are no longer associated with increases in number of clones (Figure S2).

### Infection diversity and subsequent clinical malaria

In total 36, 13 and 12 clinical episodes with fever (>37.5°C) and *P. falciparum* (>2500 parasites/µl) were reported in 337, 360 and 288 children in the follow-up periods without treatment (after surveys 1, 3 and 4), while 59 episodes were reported in 360 children during the three months after treatment (after survey 2). Age was associated with a decreased risk of clinical malaria both without and with parasite clearance before follow-up (Table 2). Children using ITN were at reduced risk after clearance (Table 2). Anemia (hemoglobin level <8 g/dl) was not associated with subsequent risk of clinical malaria (HR = 0.78, 95% CI 0.3–2.1, *P* = 0.7) after adjusting for MOI, village and age.

**Table 2:** Factors associated with risk of subsequent clinical malaria during follow-up with and without anti-malarial treatment after survey.

		No treatment after survey^a^		Treatment after survey^b^	
		Hazard ratio (95% CI)	*P*	Hazard ratio (95% CI)	*P*
*msp2* clones	0	0.47 (0.22–0.98)	0.045	0.52 (0.27–0.99)	0.045
	1*	1.0		1.0	
	≥2	1.15 (0.60–2.19)	0.67	0.46 (0.23–0.91)	0.026
Age		0.81 (0.66–0.98)	0.027	0.76 (0.63–0.92)	0.006
High transmission		0.69 (0.41–1.18)	0.18	0.82 (0.48–1.41)	0.48
ITN		1.01 (0.58–1.77)	0.97	0.35 (0.17–0.75)	0.007

a Pooled data from surveys 1, 3 and 4.

b Data from survey 2.

*Reference group.

When time to the first or only clinical episode was assessed for the respective follow-up periods, the number of clones was significantly associated with disease risk only after treatment in survey 2. There were fewer episodes in the surveys without drug-treatment, but no statistical evidence of interactions between survey number and village (*P* = 0.28), ITN use (*P* = 0.59), number of clones (*P* = 0.65) or age (*P* = 0.07). Data from these different follow-up periods without treatment (i.e. for survey 1, 3 and 4) were therefore pooled.

**Figure 3:**
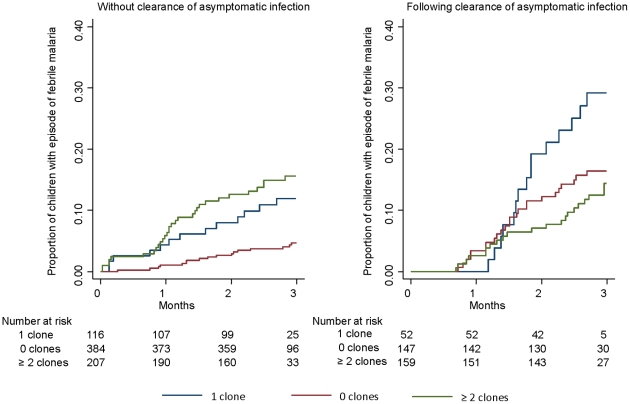
Kaplan Meier estimate of time to subsequent clinical episode without and with treatment following survey.

Children who were parasite negative at the cross-sectional surveys had a lower risk of subsequent malaria both in the follow-up periods without and with treatment; HR 0.47 (95% CI 0.22–0.98, *P = 0.045* by Cox regression) and HR 0.52 (95% CI 0.27–0.99, *P = 0.045*), respectively. The number of clones at the surveys not followed by treatment was not associated with risk compared to one clone (HR = 1.15 95% CI 0.60–2.19, *P = 0.68*) (Figure 3). However, children infected with ≥2 clones had a clearly reduced risk in the period after clearance; HR 0.46 (95% CI 0.23–0.91, *P = 0.026*) (Figure 3).

The interaction term between the number of clones and the effect of treatment on subsequent clinical malaria was analyzed in a model using data from all follow-up periods (without and with treatment). There was no significant interaction with the effect of 0 clones, HR = 0.63 (95%CI 0.24–1.66, *P = 0.36* by Cox regression). However, the interaction term was HR = 3.54 (95%CI 1.4–9.1, *P = 0.009*) for the effect of ≥2 clones and the effect of treatment. This confirms that the number of clones acts significantly differently on the risk of clinical malaria depending on whether not treatment was given after the survey.

**Figure 4:**
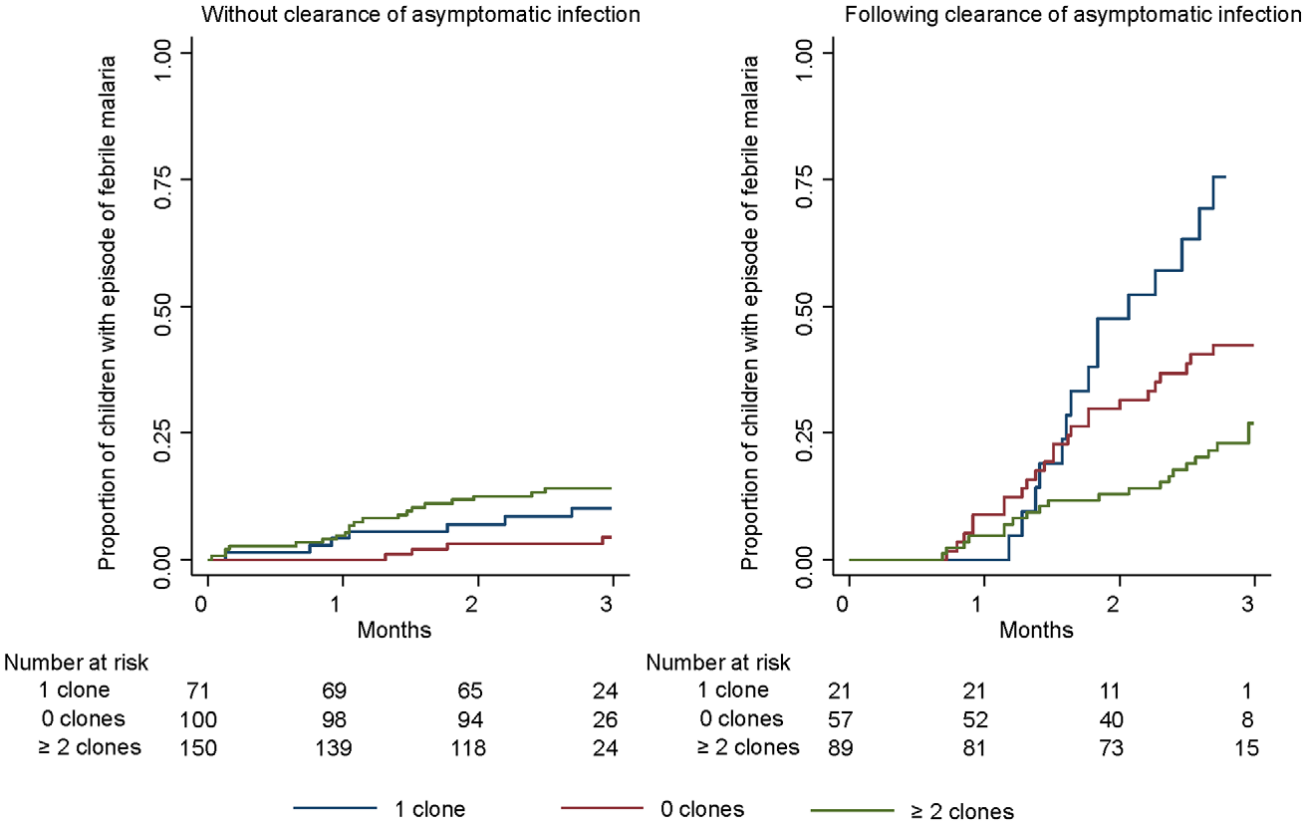
Kaplan Meier estimate of time to subsequent clinical episode with treatment excluding children who remained uninfected during follow-up.

In an analysis to distinguish the effects of lack of exposure from protection due to an efficient immunity, outcomes after treatment were analyzed in all children at the risk of re-infection (i.e. both clinical malaria and asymptomatic) compared with those remaining uninfected. In those re-infected, the risk of clinical malaria was compared with asymptomatic infections. The risk of re-infection when comparing 0 clones with 1 clone was not significantly different, however ≥2 clones was associated with an increased risk of re-infection with borderline significance (OR = 1.97 95% CI 0.99–3.93 by logistic regression) (Table 3). When clone number was used as a continuous variable, the risk of re-infection associated with clone number was OR = 1.29 (95%CI 1.06–1.58, *P = 0.013*). In those re-infected, being parasite negative or infected with ≥2 clones at survey 2 were both associated with a reduced risk of clinical malaria compared with 1 clone (OR = 0.19 95 CI 0.05–0.73 and OR = 0.06 95% CI 0.02–0.25, respectively) (Figure 4).

**Table 3:** Factors associated with re-infection, asymptomatic parasiteamia or clinical malaria during follow-up after anti-malarial treatment.

		Re-infected vs uninfected		Clinical malaria vs asymptomatic infection	
		Odds ratio (95% CI)	*P*	Odds ratio (95% CI)	*P*
*msp2* clones	0	1.10 (0.55–2.19)	0.795	0.19 (0.05–0.73)	0.015
	1*	1.0		1.0	
	≥2	1.97 (0.99–3.93)	0.053	0.06 (0.02–0.25)	<0.001
Age		0.97 (0.84–1.13)	0.301	0.51 (0.37–0.71)	0.001
High transmission		2.23 (1.40–3.56)	0.001	0.14 (0.06–0.36)	0.056
ITN		0.59 (0.35–1.02)	0.058	0.42 (0.14–1.26)	0.122

*Reference group.

## Discussion

Clearance of asymptomatic *P. falciparum* infections with one full course of an effective anti-malarial drug affected the genetic diversity of infections during follow-up and the subsequent risk of clinical malaria. Without treatment, we did not observe any significant effect of the number of clones on the risk of infection. However, when the infections were cleared, children with 2 or more clones just prior to treatment had significantly lower risk of subsequent febrile malaria than children with 1 clone. We tested the interaction term between number of clones and time of survey, and confirmed that multiclonal infections act differently depending on whether anti-malarial treatment had been given or not. Children who were parasite negative at the cross-sectional surveys had a lower risk of subsequent malaria in the following three months periods irrespective of whether the child had received anti-malarial treatment.

We analyzed the outcomes following clearance of asymptomatic infection to distinguish lack of exposure from immunity, as previously described [20]. Increasing clone number was associated with increasing risk of subsequent exposure to parasites, and the presence of 2 or more clones was associated with more blood stage immunity (i.e. less febrile malaria compared with asymptomatic infection). However, 0 clones (i.e. parasite negative), were also strongly associated with more blood stage immunity. The likelihood of being re-infected after clearance was not different from that in children with parasites, suggesting that these children experience similar levels of exposure. Being parasite negative and able to control new infections without developing symptoms thus suggests a rather efficient immunity, and ability to suppress parasites below detection levels.

The present study provides with interesting insights into the importance of multiclonal infections. Previous studies have shown conflicting results, with the number of clones being related to risk as well as to protection [13]–[15], [25], [26]. Although these differences suggest an exposure dependent component, it has not been clear whether the presence of the parasites *per se* are important or it they just represent a marker of previous exposure. In two other areas in Kilifi, infections with 2 clones were associated with an increased risk of subsequent malaria in an area of low-moderate transmission, while in a low transmission village the diversity did not predict the risk of clinical malaria [15]. Here, in this area of moderate transmission, multiclonal infections were not associated with disease risk, however when the asymptomatic infections were cleared, the risk was reduced. This might indicate an immunosuppressive effect of persistent asymptomatic infection [27]; but also that the multiclonal infections appear to have induced immunity that protect against novel infections.

Several factors can affect the number of clones in an individual at a certain time point [28]. The true number of genetically distinct parasites in an infection is likely to be underestimated due to within-host dynamics of circulating clones as well as methodological limitations. The method used for genotyping is well established in molecular epidemiological studies and the *msp2* gene has proven to be the most informative marker of parasite population diversity [29]. Moreover, fragment size determination by capillary electrophoresis used here provide high resolution and improved estimation of number and types of clones in an infection compared to previous gel based methods [19].

The number of clones was associated with age, village of residence and hemoglobin levels, however not with ITN use. There was a substantial variance in the number of clones which was not explained by age. The number of infecting clones at a certain time point might reflect the individuals' level of acquired immunity as a result of previous exposure. Children with asymptomatic, presumably chronic multiclonal infections, persisting through the preceding low transmission season may have mounted immune protection against super-infecting parasite clones while children with previous single clone infections are more susceptible when infected with novel parasite clones as the transmission season starts.

Repeated clearance of asymptomatic parasitemias is the basis of intermittent preventive treatment (IPT), a new strategy for malaria control. Genotyping of *P. falciparum* infections after 6 months of IPT given monthly or bimonthly to children in an area of high seasonal transmission in Ghana resulted in a temporary reduction of the number of clones and higher susceptibility compared to the placebo group in which ≥2 clones conferred a reduced risk [30]. IPT strategies include repeated dosage and long half life drugs with a prophylactic effect, e.g. sulphadoxine-pyrimethamine (SP) or artesunate plus amodiaquine (AS+AQ). Here a short acting drug, with no or negligible prophylactic effect, was used and thus allowed for the assessment of clearance of parasites without affecting exposure which could not be distinguished in the above study. Although performed under different transmission intensities, both studies suggest that the exposure and persistence of multiclonal infections are importance for maintenance of protective immunity.

Long periods of malaria chemoprophylaxis have been followed by increased incidence of clinical malaria [31], [32] as well as altered cellular and humoral anti-parasitic immune responses [33], [34] suggesting impaired acquisition or maintenance of clinical immunity. Antibody responses to *P. falciparum* are indeed more long lived in the presence of asymptomatic infections [6] and have been associated with a reduced risk of subsequent disease only in parasite positive children in some studies [35], [36].

In conclusion, a course of treatment of a short acting anti-malarial drug affected the diversity of *P. falciparum* infections and risk of subsequent clinical malaria. Why being infected with multiple clones is only associated with protection following clearance of parasites in this setting remains unclear. Possibly the presence of parasites is partly immunosuppressive during some stage of immune acquisition. However, multiclonal infections also represent a marker of acquired immunity and memory. Understanding how immunity to multiclonal *P. falciparum* infections, develops and how it is affected by different interventions will be important in the development and evaluation of future strategies for malaria control.

## Supporting Information

Figure S1 Correlation between age and number of clones.
(TIFF)

Figure S2 Correlation between parasite densities and number of clones.
(TIFF)

Table S1 A–C. Number of children infected with different number of clones in consecutive pairs of surveys.
(DOCX)
