# Supplementary figures and images for: Clearance of Asymptomatic P. falciparum Infections Interacts with the Number of Clones to Predict the Risk of Subsequent Malaria in Kenyan Children
Source: PLoS One. 2011 Feb 24;6(2):e16940. doi: 10.1371/journal.pone.0016940 (PMC3044709; doi:10.1371/journal.pone.0016940)

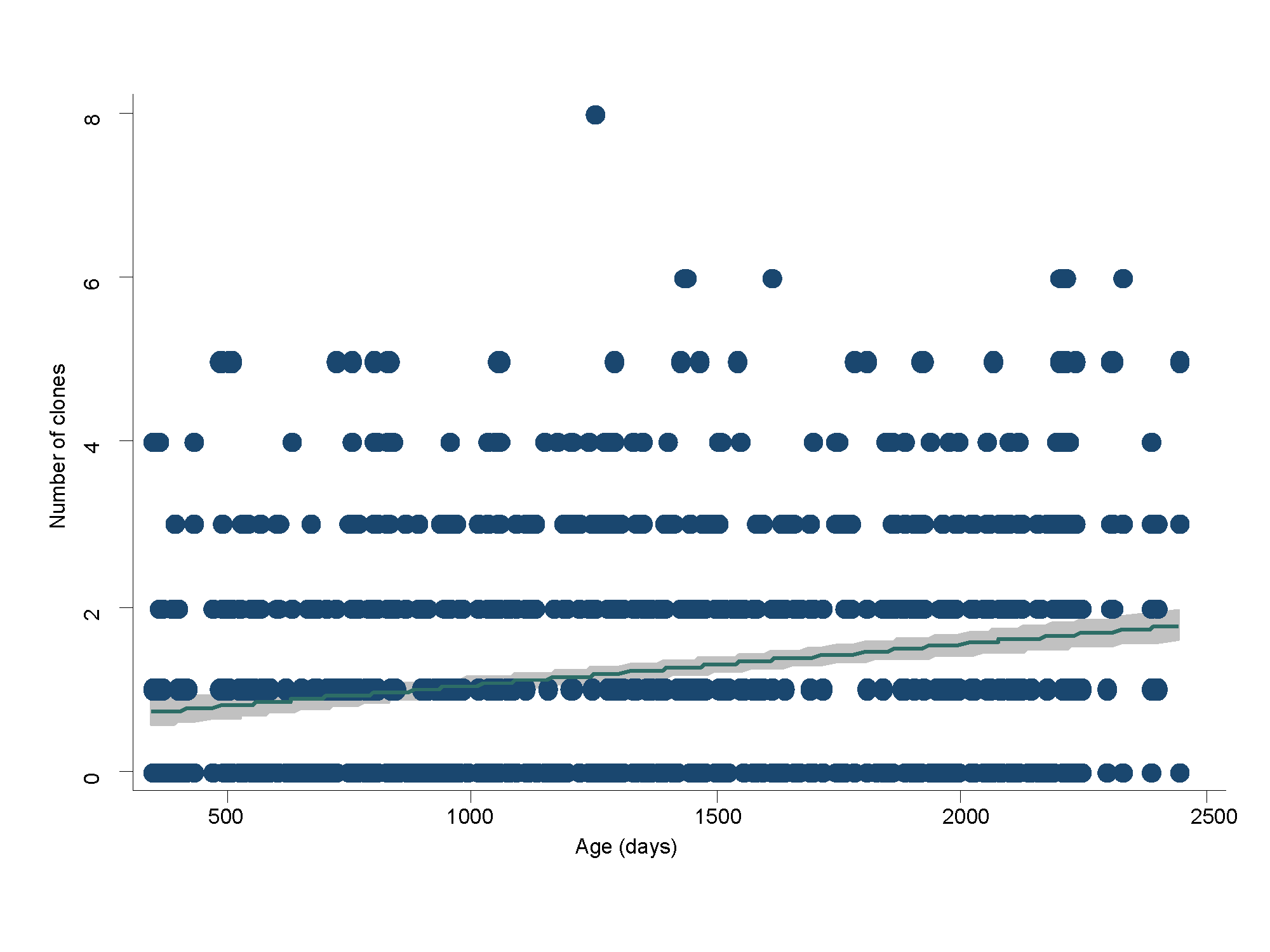

Supplement: Figure S1 — Correlation between age and number of clones. (TIFF) [file pone.0016940.s001.tiff]

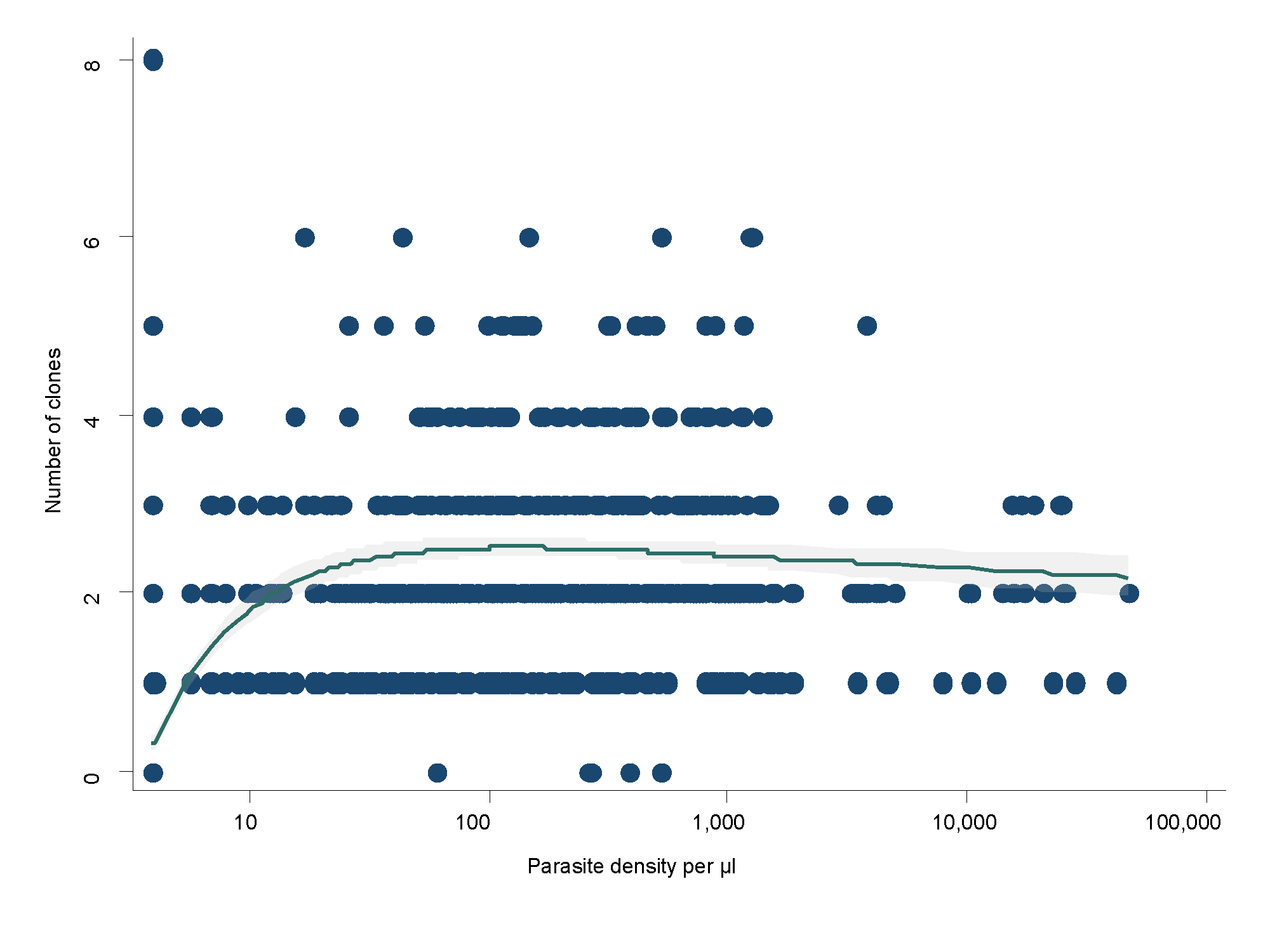

Supplement: Figure S2 — Correlation between parasite densities and number of clones. (TIFF) [file pone.0016940.s002.tiff]
