# Supplementary material for: Clearance of Asymptomatic P. falciparum Infections Interacts with the Number of Clones to Predict the Risk of Subsequent Malaria in Kenyan Children
Source: PLoS One. 2011 Feb 24;6(2):e16940. doi: 10.1371/journal.pone.0016940 (PMC3044709; doi:10.1371/journal.pone.0016940)
Supplement: Table S1 — A–C. Number of children infected with different number of clones in consecutive pairs of surveys. (DOCX) [file pone.0016940.s003.docx]

**Table S1. Number of children infected with different number of clones in consecutive pairs of surveys**

**A**

|  | **2^nd^ survey** |  |  |  |  |  |
| --- | --- | --- | --- | --- | --- | --- |
| **1^st^ survey** | **0** | **1** | **2** | **3** | **4** | **5** |
| **0** | 46 | 11 | 8 | 2 | 0 | 0 |
| **1** | 27 | 15 | 12 | 5 | 2 | 1 |
| **2** | 23 | 11 | 26 | 11 | 2 | 2 |
| **3** | 10 | 5 | 19 | 11 | 9 | 2 |
| **4** | 8 | 1 | 4 | 7 | 0 | 2 |
| **5** | 5 | 0 | 3 | 2 | 0 | 1 |
| **6** | 1 | 0 | 1 | 1 | 1 | 0 |
| **8** | 0 | 0 | 1 | 0 | 0 | 0 |

**B**

|  | **3^rd^ survey** |  |  |  |  |  |
| --- | --- | --- | --- | --- | --- | --- |
| **2^nd^ survey** | **0** | **1** | **2** | **3** | **4** | **5** |
| **0** | 77 | 16 | 14 | 1 | 3 | 0 |
| **1** | 30 | 6 | 3 | 1 | 0 | 0 |
| **2** | 35 | 14 | 9 | 3 | 1 | 1 |
| **3** | 13 | 5 | 8 | 1 | 1 | 0 |
| **4** | 7 | 0 | 5 | 1 | 2 | 0 |
| **5** | 2 | 0 | 3 | 1 | 0 | 0 |

**C**

|  | **4^th^ survey** |  |  |  |  |  |  |
| --- | --- | --- | --- | --- | --- | --- | --- |
| **3^rd^ survey** | **0** | **1** | **2** | **3** | **4** | **5** | **6** |
| **0** | 102 | 14 | 13 | 5 | 0 | 1 | 0 |
| **1** | 10 | 11 | 8 | 2 | 1 | 0 | 0 |
| **2** | 8 | 5 | 8 | 5 | 4 | 1 | 2 |
| **3** | 0 | 0 | 3 | 2 | 1 | 0 | 0 |
| **4** | 0 | 0 | 1 | 0 | 0 | 0 | 0 |
| **5** | 2 | 0 | 3 | 1 | 0 | 0 | 0 |
